# Effects of secondary metabolites produced by different cyanobacterial populations on the freshwater zooplankters *Brachionus calyciflorus* and *Daphnia pulex*

**DOI:** 10.1007/s11356-019-04543-1

**Published:** 2019-02-27

**Authors:** Barbara Pawlik-Skowrońska, Magdalena Toporowska, Hanna Mazur-Marzec

**Affiliations:** 1 Department of Hydrobiology and Protection of Ecosystems, University of Life Sciences in Lublin, Dobrzańskiego 37, 20-262 Lublin, Poland; 2 Division of Marine Biotechnology, University of Gdańsk, al. Piłsudskiego 46, 81-378 Gdynia, Poland

**Keywords:** Microcystins, Anatoxin-a, Anabaenopeptins, Cyanopeptolins, Zooplankton

## Abstract

Cyanobacterial blooms in eutrophic water bodies are a worldwide problem. Combined effects of mixtures of secondary metabolites produced by different cyanobacterial species on aquatic fauna are still not well recognised. We compared the survivorship of *Brachionus calyciflorus* Pallas (Rotifera) and *Daphnia pulex* Leyding (Cladocera) exposed to pure microcystin LR (MC-LR), anatoxin-a (ANTX) and to five extracts obtained from bloom-forming cyanobacteria *Microcystis*, *Planktothrix* and *Dolichospermum.* The obtained results revealed different response of the organisms to high concentrations of pure MC-LR, ANTX and complex cyanobacterial extracts. The extracts’ toxicity to invertebrates was higher than that exerted by pure cyanotoxins and was dependent on the composition of cyanobacterial metabolites: *Microcystis* spp. extract containing anabaenopeptins A and B, aeruginosamide, four variants of cyanopeptolins and five MCs was not toxic to either of the organisms, whereas *Planktothrix agardhii* extract (I), containing anabaenopeptins A, B, F, 915, oscillamide Y, five different aeruginosins and four variants of MC was more toxic to daphnids than to rotifers. The extracts of another *P. agarhdii* (II) biomass and two different biomass samples of *Dolichospermum* spp. also affected survivorship of the rotifer and cladoceran, however, to various extent. It strongly suggests that non-ribosomal oligopeptides, other than MCs, had essential contribution to the observed toxicity to invertebrates and their effects on particular species or populations can vary depending on the secondary metabolite profiles of cyanobacteria.

## Introduction

Water blooms caused by cyanobacteria are an increasing global problem. Many of the bloom-forming species produce various toxins such as microcystins (MCs), anatoxin-a (ANTX), anatoxin-a(S) (ANTX-S), saxitoxins (STX) and other metabolites harmful to aquatic organisms, including zooplankton species (Ferrão-Filho and Kozlowsky-Suzuki 2011; Metcalf and Codd 2012; Toporowska et al. 2014; Pearson et al. 2016; Bownik 2016; Osswald et al. 2007). In freshwaters affected by cyanobacterial blooms, replacement of larger planktonic invertebrates (e.g. *Daphnia*; Cladocera) by smaller cladocerans (e.g. *Bosmina*), rotifers and copepods has been reported (Leonard and Pearl 2005; Hansson et al. 2007). According to published reports, this phenomenon may be a consequence of (i) a reduction of the filtering efficiency of larger zooplankton species by filaments and colonies of cyanobacteria (DeMott et al. 2001); (ii) a deterioration of the quality of algal food (Tillmanns et al. 2008); and/ or (iii) harmful effects of cyanobacterial metabolites on different zooplankton genera and species (Hulot et al. 2012; Smutná et al. 2014). These effects, however, have been insufficiently recognised, yet.

Studies on cyanobacteria-zooplankton interactions have shown diverse and sometimes contradictory results (Tillmanns et al. 2008; Ger et al. 2014 and references therein). Most reports concern cell-bound (intracellular) compounds or effects of a single extracellular metabolite, especially MCs. For example, MC-producing *Microcystis* spp*.* as a diet inhibited growth rate, reproduction and lifespan of some Cladocera (*Daphnia*, *Moina* or *Ceriodaphnia*) and Rotifera (Lürling 2003; Geng and Xie 2008; Han et al. 2012). Zooplankton accumulates cyanotoxins by intestinal uptake and ingestion of cyanobacteria (Rohrlack et al. 2005), however, it can be also affected by toxins dissolved in water. Therefore, an increasing attention has been paid to the influence of the dissolved fraction of cyanobacterial metabolites on aquatic organisms, including zooplankton (Smutná et al. 2014; Ferrão-Filho et al. 2014; Barrios et al. 2015). During cyanobacterial mass development or cell lysis, especially during bloom collapse, a mixture of toxins and other cyanobacteria cell components occurs in water and can achieve high concentrations. For example, in a Polish lake with *P. agardhii* bloom 11 μg/L of dissolved MCs was found (Pawlik-Skowrońska et al. 2008), but much higher cyanotoxin concentrations (even up to 226.2 μg MCs/L and up to 126 μg cylindrospermopsin/L; Messineo et al. 2009) were determined in Italian freshwaters. During *Microcystis* bloom collapse, the concentrations of dissolved MCs reached up to 712 μg/L (Nasri et al. 2004) and even up to 1800 μg/L after treatment of bloom with an algicide (Jones and Orr 1994). The influence of cyanobacterial metabolites (other than known toxins) on zooplankton reaches an increasing interest (Blom et al. 2006; Czarnecki et al. 2006; Schwarzenberger et al. 2013a; Kohler et al. 2014) due to their inhibitory activity to some enzymes such as serine proteases, protein phosphatases, carboxypeptidases. Oligopeptides constitute a very large group of cyanobacterial products such as many variants of anabaenopeptins, aeruginosins and microginins (Lifshits and Carmeli 2012) commonly occurring in eutrophic water bodies at different concentrations. As reported by Schwarzenberger et al. (2013b), production of serine protease inhibitors such as micropeptins by *M. aeruginosa* was dependant on biogenic compounds (N and P) availability, which also increases cyanobacterial development in nutrient-rich waters. Ecological and biological role of oligopeptides still remains unclear (Baumann and Jüttner 2008; Agha and Quesada 2014), albeit negative influence of some of them on aquatic organisms has been reported (Blom et al. 2006; Czarnecki et al. 2006; Kohler et al. 2014). We agree with the statement of Ger et al. (2014), that since cyanobacteria produce more than one bioactive metabolite, the unsystematic designation of toxicity based on a single well-identified compound is insufficient to assess the environmental impact of cyanobacterial bloom and should be revised. Therefore, the effects of complex mixtures of cyanobacterial secondary metabolites on zooplankton (and other aquatic organisms) should be thoroughly studied, what was recently strongly emphasised by Barrios et al. (2015).

The aim of this work was to compare the effect of pure cyanotoxins (MC-LR, ANTX) and complex mixtures of metabolites produced by different populations of bloom-forming cyanobacterial genera on the survivorship of two freshwater zooplankters of worldwide distribution. We also hypothesised that the non-ribosomal oligopeptides (other than MCs) produced by different cyanobacteria may essentially contribute to their toxicity to zooplankton.

## Materials and methods

### Sampling and identification of cyanobacteria

As indicated in Table 1, four samples of surface scum formed by the coccoid *Microcystis* spp. or *Dolichospermum flos-aquae* (Brébisson ex Bornet & Flahault) P. Wacklin, L. Hoffmann and J. Komárek or *D. lemmermannii* (Ricter) P. Wacklin, L. Hoffmann and J. Komárek, or *Planktothrix agardhii* (Gomont) Anagnostidis and Komárek and one sample of *P. agardhii* bloom concentrated with a plankton net (25 μm) were collected in the following spring-autumn seasons in three lakes—Piaseczno Lake (one sample), Wytyckie Lake (one sample) and Syczyńskie Lake (three samples) (E. Poland). The lakes differ in trophic status: from mesotrophic to hypertrophic, respectively. Taxonomic identification of cyanobacteria was carried out according to Komárek and Anagnostidis (1999, 2000, 2005) and Wacklin et al. (2009).

**Table 1:** Characteristics of the cyanobacterial biomasses sampled in the lakes and their crude extracts used in bioassays

Lake	Species composition	Extract code	Chl-a (mg/L)	Total MCs (mg/L)	ANTX (mg/L)	Toxins (MCs + ANTX) to Chl-a ratio
Wytyckie	*Microcystis aeruginosa* (90%) *M. natans* (5%) *M. wesenbergii* (5%)	A	7.90 ± 0.24	5.00 ± 0.14	n.d.	0.633
Syczyńskie	*Planktothrix agardhii* I (97%) *Aphanizomenon gracile* (2%), *Dolichospermum* spp. (1%)	B	29.66 ± 0.07	14.80 ± 0.35	n.d.	0.499
Syczyńskie	*P. agardhii* II (99%), *A. gracile* (1%)	C	115.51 ± 13.65	5.46 ± 0.73	n.d.	0.047
Syczyńskie	*Dolichospermum flos-aquae* (98%), *A. gracile* (2%)	D	10.88 ± 2.99	0.06 ± 0.01	0.31 ± 0.01	0.0345
Piaseczno	*Dolichospermum lemmermannii* (99%), *Dolichospermum* spp. (1%)	E	70.48 ± 3.99	8.81 ± 0.65	2.05 ± 0.15	0.154

*n.d.* not detected

Data are expressed as means ± SD, *n* = 3

### Cyanobacterial biomass extraction

A total of 15 to 20 mL of each mentioned above samples of cyanobacterial biomass were used to prepare aqueous extracts for bioassays and to determine the MCs and ANTX concentrations. Fresh biomass samples were sonicated for 5 min. and after centrifugation (14,000×*g* for 10 min, 17 °C), supernatants were collected and frozen (−20 °C) until the day of cyanotoxin analysis and bioassays. Chlorophyll-a concentration (an indicator of the total cyanobacterial biomass) in the ethanol extracts was determined spectrophotometrically (at 665 and 750 nm) according to the norm PN-ISO 10260 (2002).

A total of 10 mL of *Microcystis* spp. (A) and *P. agardhii*-dominated (B) bloom samples were used to prepare extracts for LC-MS/MS analysis of cyanobacterial oligopeptides. Other cyanobacterial samples were not analysed for the detailed composition of oligopeptides other than MCs due to a lack of material. The samples were filtered onto GF/C glass-fibre filters (Whatman) and extracted with 5.0 mL of 5% acetic acid in MilliQ water by 1-min probe sonication with an ultrasonic disrupter followed by 15-min bath sonication. After centrifugation (10,000*g* for 15 min), supernatants were collected and subjected to LC-MS/MS analysis.

### HPLC-PDA analysis of microcystins

A high-performance liquid chromatography-photodiode array detection system (Shimadzu) was used for microcystin (MC) detection in cyanobacterial extracts according to Lawton et al. (1994). Microcystins were separated using the following mobile phases: A, water acidified with 0.05% trifluoroacetic acid (TFA, Merck) and B, acetonitrile (Merck) acidified with 0.05% TFA (the gradient 30–100%) at a flow rate of 0.7 mL/min in a RP-18 Purosphere column (125 × 3 mm, dp 5 μm, Merck). The wavelength range for detection was 200–300 nm. For identification and quantitative analyses, MC-LR, MC-RR, MC-YR, MC-LA, MC-LY, MC-LF, MC-WR, MC-HtyR (Alexis) were used as standards. Other MC variants were identified on the base of their specific absorption spectra, retention time and confirmed by LC-MS/MS analysis.

### HPLC-FLD analysis of anatoxin-a

ANTX was determined using HPLC with fluorescence detection (FLD, Beckman). Ten microliters of each extract was reconstituted with 100 μl of 0.1 M sodium borate in a 2-mL Eppendorf probe. Fifty microliters of 10% NBD-F (4-fluoro-7-nitrobenzofuran; Fluka) in acetonitrile was added and the mixture was allowed to stand (10 min) in the dark at room temperature. Fifty microliters of 1 M HCl was added to terminate the reaction and HPLC-FLD was performed using an RP-18 Purospher column (125 × 3 mm, 5 μm, Merck) at 25 °C. The mobile phases were A, water acidified with 0.05 % trifluoroacetic acid (TFA, Merck) and B, acetonitrile (Merck) acidified with 0.05% TFA (45:55), at a flow rate of 0.6 mL/min. The detector parameters were as follows: excitation wavelength 470 nm, emission wavelength 530 nm. ANTX standard (Tocris, Bioscience) was used for identification and quantitative determinations of the toxin.

### LC-MS/MS analysis of oligopeptides

Structural analyses of cyanobacterial peptides were performed using Agilent 1200 (Agilent Technologies, Waldboronn) coupled online to a hybrid triple quadrupole/linear ion trap mass spectrometer (QTRAP5500, Applied Biosystems, Sciex; Concorde, ON). As a mobile phase, a mixture of A (5% acetonitrile in MilliQ water plus 0.1% formic acid) and B (0.1% formic acid in acetonitrile) was used. Separation was performed on a Zorbax Eclipse XDB-C18 column (4.6 × 150 mm; 5 μm) (Agilent Technologies, Santa Clara, CA). Phase B was linearly increased from 15 to 75% in 5 min and then to 90% in the next 5 min. This composition of the mobile phase was held for 5 min and brought back to 15% B in 1 min. The column oven temperature was 35 °C, the flow rate was 0.6 mL/min and the injection volume was 5 μL. The turbo ion spray (550 °C) voltage was 5.5 kV, with the nebuliser gas pressure and curtain gas pressures set at 60 p.s.i. and 20 p.s.i., respectively.

To characterise the structure of cyanobacterial peptides with the MS/MS system, the experiments were run using the information-dependent acquisition method (IDA) and in enhanced ion product mode (EIP). In EIP mode, ions fragmented in the collision cell (Q2) were captured in the ion trap and then scanned. In the IDA method, Q3 survey scans were used to automatically trigger an EIP scan if the signal was above a threshold of 500,000 cps. EPI spectra were acquired from 50 to 1000 Da with a scan speed of 2000 Da s^−1^ and a collision energy (CE) of 45 V with a collision energy spread (CES) of 20 V. The linear ion trap fill time was 50 ms. Dynamic exclusion was activated to minimise the risk of missing the co-eluting compounds. Data acquisition and processing were done using Analyst QS® 1.5.1 software. Relative contribution of particular oligopeptides in their total amount was estimated on the basis of peak area of the pseudo-molecular ions.

### Acute toxicity bioassays

The toxicity of MC-LR (Alexis), ANTX (Torcis, Bioscence) and five aquatic extracts of different cyanobacterial bloom samples containing MCs or both MCs and ANTX as well as other oligopeptides towards the juvenile freshwater rotifer *Brachionus calyciflorus* Pallas (Rotoxkit F) and the cladoceran *Daphnia pulex* Leyding (Daphtoxkit F^TM^ pulex) was evaluated in 24-h bioassays. In each replicate, five specimen of *B. calyciflorus* or *D. pulex* were exposed in 0.3 mL or 1 ml of standard medium, respectively. The assays were performed under the same laboratory conditions (temperature 20^°^C, in darkness), according to the producer (Microbiotests, INC, Gent, Belgium) protocols using different concentrations of MC-LR or ANTX and a range of dilutions of cyanobacterial extracts in a standard medium. The biomass of the extracted cyanobacterial materials was expressed as chl-a concentrations and ranged in the tests from 0.16 to 6.66 mg Chl-a/L. Four dilutions of each extract of *Microcystis* spp. (A), *P. agardhii* I (B) and *P. agardhii* II (C) containing MCs, and four dilutions of each extract of *D. flos-aquae* (D) and *D. lemmermannii* (E) containing both MCs and ANTX, were tested. The assays were performed three times, in three replicates. The test end-point was the death of the organisms. As controls, in the tests with the extracts and ANTX, the organisms were incubated in the standard medium. The influence of 0.5% methanol (a maximum concentration used as a solvent for pure MC-LR only) on the survivorship of rotifers and cladocerans was also examined and no toxic effect was observed.

### Toxicity data analysis and statistical analysis

Toxicity data obtained in the bioassays were expressed as the percentage (%) of the organisms’ survivorship as compared to the controls. Data were expressed as means ± standard error of the mean (SEM). Significant differences among treatments were evaluated using one-factor analysis of variance (ANOVA). Pair-wise comparison of means was done using the Tukey test (*p* < 0.05). For determination of the acute toxicity parameter (24-h LC50), Probit analysis was used. LC50 values with non-overlapping 95% intervals were regarded as being significantly different. Correlations between the concentrations of pure MC-LR, ANTX or extracts and the survivorship of rotifers and daphnids were calculated using the Pearson coefficient at *p* < 0.05.

## Results

### Differences in secondary metabolites produced by cyanobacteria

The extracts obtained from different biomasses (7.90–115.51 mg Chl-a/L) of bloom-forming cyanobacteria contained high total concentrations of MCs only (5.00–14.80 mg/L, extracts A, B and C) or both MCs and ANTX together (0.37–10.86 mg/L, extracts D and E) but in different proportions (Table 1). The biomasses of *Microcystis* spp. (A) and *P. agardhii* I (B), sampled from different lakes were characterised by high ratio of the total MC content to Chl-a (0.633 and 0.499, respectively). They were additionally analysed for oligopeptides other than MCs (Table 2). The extract A (*Microcystis* spp.) contained five MCs and seven other oligopeptides (mainly cyanopeptolins). The extract B (*P. agardhii* I) contained four MCs and 11 other oligopeptides (anabaenopeptins and aeruginosins). In other three cyanobacterial biomasses, only ANTX and MCs, due to a lack of material, were analysed. On the basis of the signal intensity of pseudo-molecular ions (data not shown), it was concluded that out of the detected metabolites, the relative content of MC-LR and [Asp^3^]MC-HarR in extract A was the highest followed by anabaenopeptin A (AP A) and aeruginosamide (AERMD). Three other MC variants, anabaenopeptin B and four variants of cyanopeptolins were also present (Table 2). In extract B, from the biomass of *P. agardhii* I (97%) with an inessential admixture of some Nostocales, two derivatives of MC-RR, [Asp^3^dha^7^]MC-LR, five anabaenopetins, aeruginosin 89 and five aeruginosides had similarly high relative contribution (Table 2). In extract C, obtained from about fourfold higher biomass (115.51 mg Chl-a/L) of *P. agardhii* II (99%; Table 1), in which only MCs were detected, the desmethyl derivative of MC-RR predominated over two other MC variants (data not shown). However, the total MC concentration in the *P. agardhii* II extract (MCs to Chl-a ratio 0.047) was about threefold lower than in the case of extract B from the *P. agardhii* I biomass sampled from the same lake but in different season. Extract D, obtained from *D. flos-aquae* (98% of biomass) and *Aph. gracile* (2%), contained mainly ANTX (0.31 mg/L) and fivefold lower concentration of MCs (MC-LY and two unidentified MC variants). Altogether, in the extract D cyanotoxins (MCs and ANTX) to Chl-a ratio was 0.0345 (Table 1). Extract E, from *D. lemmermannii* (99% of biomass) and other *Dolichospermum* spp. (1%), contained much higher total concentration of microcystins (8.81 mg/L) than the extract D, with nine different MC variants (MC-LA, MC-LR, MC-YR, MC-LW, MC-LF and four unidentified) and ANTX (2.05 mg/L). Cyanotoxins to Chl-a ratio in the extract was 0.154.

**Table 2:** MC variants and other oligopeptides identified by means of LC-MS/MS in two cyanobacterial extracts used in bioassays

MC variants	[M + H]^+^*m*/*z*	Extracts	
		*Microcystis* spp. (A)	*P. agardhii* (B)
[Asp^3^]MC-HtyR	1045	++	++
[Asp^3^]MC-HarR	1038	+++	
[Ser^7^]MC-RR	1042		+++
[Asp^3^Mdha^7^]MC-RR	1024		+++
MC-LR	995	+++	
[Asp^3^]MC-LR	981	+	
[Asp^3^dha^7^]MC-LR	981		+++
MC-LY	1002	+	
Other oligopeptides			
Anabaenopeptin A	844	+++	+++
Anabaenopeptin B	837	++	+++
Anabaenopeptin 915	916		+++
Anabaenopeptin F	851		+++
Oscillamide Y	858		+++
Aeruginosamide	561	+++	
Cl-aeruginoside 126	749		+++
Aeruginoside 716	717		+++
Aeruginoside 126 A	715		+++
Aeruginoside 126 B	691		+++
Aeruginosin 89	637		+++
Dechloro-aeruginosin 89	603		++
Cyanopeptolin CPL997	998	+	
Cyanopetolin CPL917	918	+	
Cyanopeptolin CPL863	863	++	
Cyanopeptolin CPL827	828	++	

Relative contribution of particular peptides found in extracts based on signal intensity. (Counts per second) cps × 10^6^ low (+); × 10^7^ medium (++); × 10^8^ high (+++)

### Effect of pure cyanotoxins on zooplankton

**Fig. 1:**
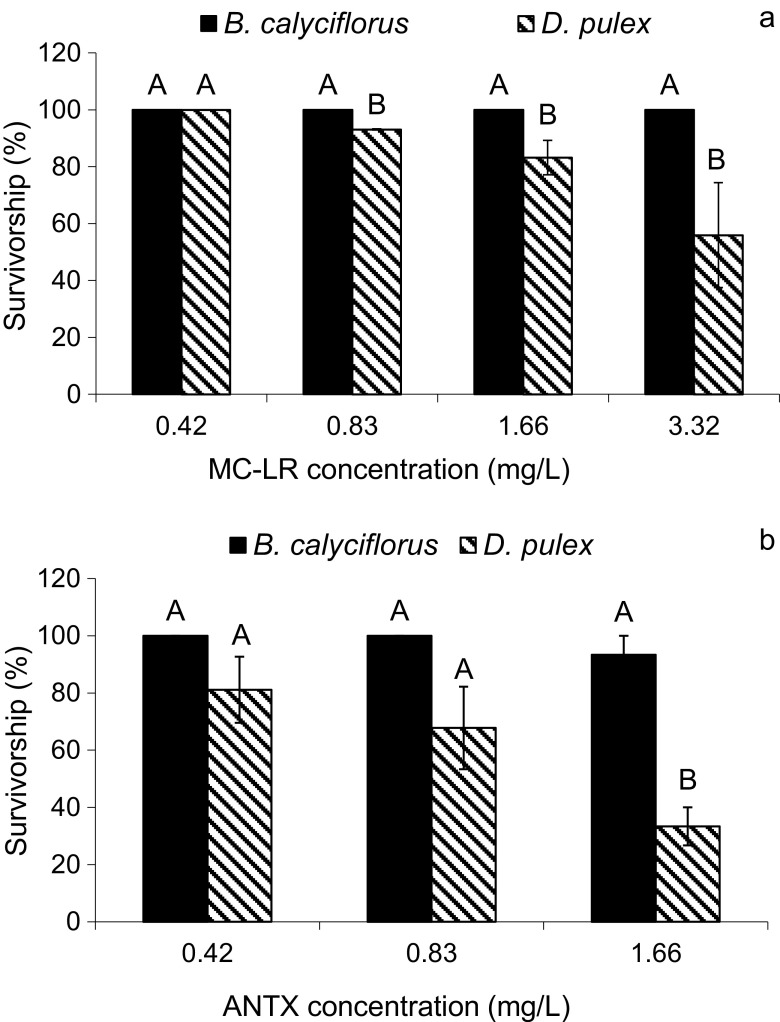
The influence of pure MC-LR (**a**) and ANTX (**b**) on the survivorship of *B. calyciflorus* and *D. pulex* (mean ± SEM; *n* = 9). The survivorship of organisms in controls was set as 100%. Different uppercase letters (A, B) indicate statistically significant differences between the survivorship of zooplankters exposed to different concentrations of MC-LR (**a**) and ANTX (**b**) (ANOVA, Tukey’s test, *p* < 0.05)

High concentrations (1.66–3.32 mg/L) of pure MC-LR (Fig. 1a) caused an acute toxic effect on *D. pulex*, only (Fig. 1a, Table 4). However, for *D. pulex*, the 24-h LC50 value of MC-LR seems to be higher than 3.32 mg/L, and it was impossible to determine it within the concentration range used. For *B. calyciflorus*, MC-LR was non-toxic within the range 0.42–3.32 mg/L (Fig. 1a). The bioassays showed that *B. calyciflorus* was also more resistant than *D. pulex* to pure ANTX (Fig. 1b, Table 4). ANTX decreased significantly the survivorship of cladoceran to approx. 33% at the highest concentration used (1.66 mg/L) and the effect of ANTX was stronger than of MC-LR at the same concentrations (Fig. 1b, Table 4). The 24-h LC50 values of ANTX for *D. pulex* and *B. calyciflorus*, however, were not determined within the concentration range used.

### Effect of metabolites contained in aqueous cyanobacterial extracts on zooplankton

**Fig. 2:**
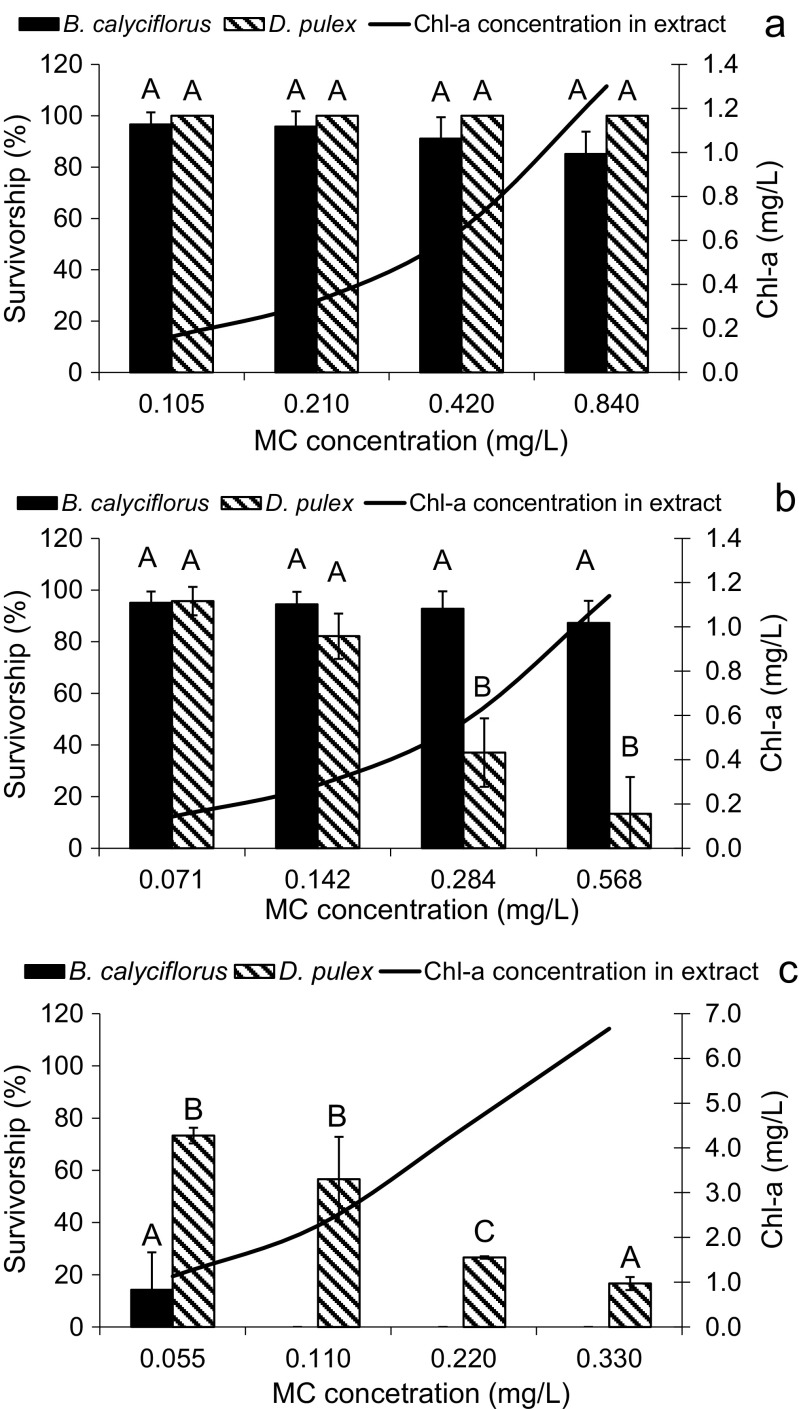
The influence of extracts of MC-producing *Microcystis* spp. (**a**), *P. agardhii* I (**b**) and *P. agardhii* II (**c**) on the survivorship of *B. calyciflorus* and *D. pulex* (mean ± SEM; *n* = 9). The survivorship of organisms in controls was set as 100%. Different uppercase letters (A, B, C) indicate statistically significant differences between the survivorship of zooplankters exposed to different dilutions of extracts of *Microcystis* spp. (**a**), *P. agardhii* I (**b**) and *P. agardhii* II (**c**) with determined cyanotoxins and Chl-a concentrations (ANOVA, Tukey’s test, *p* < 0.05)

The dilutions of the extract of *Microcystis* spp. (A) and the extract (B) of the biomass composed mainly of *P. agardhii* I (Fig. 2a, b), had similar chlorophyll-a concentrations (max, 1.3 and 1.4 mg Chl-a/L, respectively) and similar range of total MC concentrations (Fig. 2a, b), however, they differed considerably in the profile of MCs and other oligopeptides (Table 2). Toxic effects of these cyanobacterial extracts on the tested organisms were also different (Fig. 2a, b). Extract A, containing five MCs and seven other oligopeptides (with the relatively highest contribution of AP A and AERMD and lower of cyanopeptolins; Table 2), did not exert acute toxic effects neither to rotifers nor to cladocerans (Fig. 2a, Table 4). However, extract B (Table 2), containing a higher number (11) of other oligopeptides than extract A, and four other MC variants (Table 2) was not essentially toxic to rotifers but significantly decreased cladoceran survivorship (approximately to 13% of the control; Fig. 2b, Table 4).

The dilutions of extract C (from *P. agardhii* II) with higher Chl-a concentrations (max, 6.66 mg Chl-a/L) and slightly lower concentrations of MCs than extracts A and B, but an unknown content of other oligopeptides, were significantly toxic to both tested organisms, however, in a contrary to extracts A and B, much more to *B. calyciflorus* than to *D. pulex* (Fig. 2c, Tables 3 and 4). For example, in the diluted extract C containing 0.055 mg MCs/L, the survivorship of rotifers decreased on average by 83%, whereas survivorship of cladocerans was reduced by 25% in comparison with untreated organisms. The extract dilutions with several times higher MC concentrations caused 100% mortality of rotifers (Fig. 2c). The 24-h LC50 value of the extract C (based on the Chl-a concentration; Table 3) for *B. calyciflorus* (0.587 mg Chl-a/L) was approx. fourfold lower than for *D. pulex* (2.433 mg Chl-a/L).

**Table 3:** Toxicity (24-h LC50) of MCs or MCs and ANTX containing cyanobacterial extracts to *B. calyciflorus* and *D. pulex*, determined on the base of cyanobacterial Chl-a concentration used as the equivalent of the cyanobacterial biomass

Extract code	LC50 expressed as mg Chl-a/L	
	*Brachionus calyciflorus*	*Daphnia pulex*
A	n.d.	n.d.
B	n.d.	0.475 ± 0.053
C	0.587 ± 0.039	2.433 ± 0.231
D	n.d.	n.d.
E	0.774 ± 0.040	1.234 ± 0.207

*n.d*. not determined within the extract concentration range used

Data are expressed as means ± 95% confidence intervals

**Table 4:** Values of Pearson’s coefficients showing correlation between the concentrations of pure MC-LR, ANTX, cyanobacterial extracts and survivorship of *B. calyciflorus* and *D. pulex*

	*B. calyciflorus*	*D. pulex*
MC-LR	0	− 0.58**
ANTX	− 0.46	− 0.74**
A	− 0.37	0
B	− 0.30	− 0.84**
C	− 0.91*	− 0.80**
D	− 0.75**	− 0.77**
E	− 0.89**	− 0.97**

A–E, codes of extracts. Significant results in italics; *0.05 < *p* < 0.01, **0.01 < *p* ≤ 0.001

**Fig. 3:**
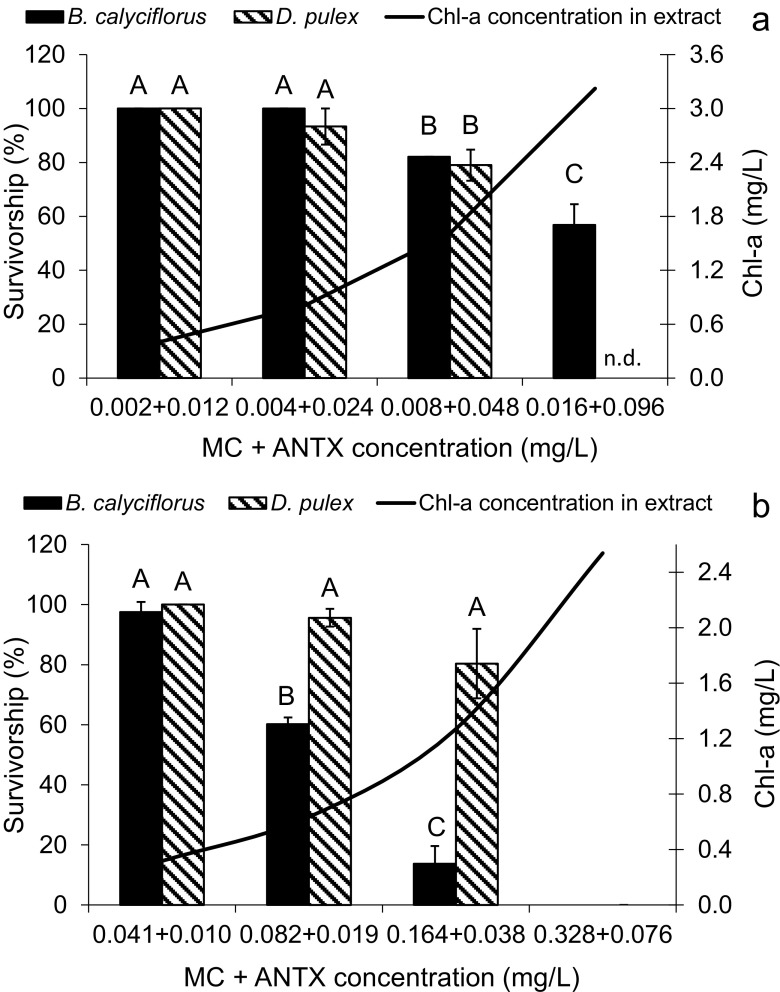
The influence of extracts of MC- and ANTX-producing *D. flos-aque* (**a**) and *D. lemmermannii* (**b**) on the survivorship of *B. calyciflorus* and *D. pulex* (mean ± SEM; *n* = 9). The survivorship of organisms in controls was set as 100%. Different uppercase letters (A, B, C) indicate statistically significant differences between the survivorship of zooplankters exposed to different dilutions of extracts of *D. flos-aque* (**a**) and *D. lemmermannii* (**b**) with determined cyanotoxins and Chl-a concentrations (ANOVA, Tukey’s test, *p* < 0.05). n.d. not determined

The extracts D and E of the biomasses of different species of *Dolichospermum* containing both MCs and ANTX (in different proportions) but unknown amounts of other oligopeptides were also significantly toxic to the organisms tested (Fig. 3a, b, Table 4). The *D. flos-aquae* extract (D), at low total cyanotoxins’ concentrations (up to 0.056 mg/L) containing sixfold higher ANTX than MCs (Fig. 3a), exerted a similar acute toxic effect on rotifers and cladocerans. As indicated by correlation coefficient (Table 4), the survivorship of rotifers and cladocerans decreased also significantly after short-term exposure to extract E from *D. lemmermannii* (Fig. 3b). The extract E was more toxic to rotifers (24-h LC50 = 0.774 mg Chl-a/L) than to cladocerans (24-h LC50 = 1.234 mg Chl-a/L). The dilutions of the extract E used in the test contained almost 21-fold higher concentrations of MCs and slightly lower ANTX concentrations than in the extract D, but affected *B. calyciflorus* more strongly than *D. pulex* (Fig. 3a, b). At the lowest dilution of the extract E from *D. lemmermannii* containing the highest cyanotoxins’ concentration, a 100% lethal effect on both organisms was observed (Fig. 3b).

## Discussion

In recent years, progressive water eutrophication and predicted climate warming have prompted interest in toxigenic cyanobacterial blooms (Rigosi et al. 2014) and their effects on the functioning of aquatic biocenoses and ecosystems (Pawlik-Skowrońska et al. 2013; Toporowska and Pawlik-Skowrońska 2014; Toporowska et al. 2014; Ger et al. 2014; Sukenik et al. 2015). Daphnids (Cladocera) and rotifers (Rotifera) play important roles in various water bodies as a part of food chains (Ger et al. 2014).

Experiments with single MC-LR and ANTX showed that very high concentrations of toxins present in water can exert acute toxic effects on zooplankton. However, in nature, aquatic organisms face the mixtures of various metabolites present both in cyanbacterial cells and in water after cell lysis (Metcalf and Codd 2012). Therefore, we compared the effects of five natural mixtures of cyanobacterial metabolites (ANTX, MCs and other oligopeptides) present in aqueous extracts of different bloom samples on different invertebrates of zooplankton communities.

Our results indicate that rotifers were, generally, more resistant than daphnids to pure toxins (ANTX and MC-LR). However, the toxicity of cyanotoxins can be species-specific and may depend on the exposure route—cyanobacterial cell ingestion and/or absorption of dissolved toxins. As previously reported by Gilbert (1994), survivorship of the rotifer *Synchaeta pectinata* Ehrenberg was already affected at 0.20 mg ANTX/L, whereas the survivorship of *D. pulex* did not decrease at 1 mg of ANTX/L; however, the day of first reproduction or interclutch interval were affected in a temperature-dependent manner. Toxicity of MCs towards daphnids has been previously reported (Wiegand and Pflugmacher 2005) and it was found that the lethal effects depend on the amount of toxin accumulated in different species and type of feeding. As reported by Tonk et al. (2005), different MC variants may reveal various toxicity. For example, more hydrophobic MC-LW, MC-LF were more toxic *in vivo* to invertebrates than MC-LR and MC-LY variants (Ward and Codd 1999). Therefore, the effects that were observed here have been a consequence of physical-chemical variation and different bioactivity of various cyanobacterial metabolites as well as sensitivity of individual species or even populations of aquatic invertebrates.

Our experiments confirmed much higher toxicity of the cyanobacterial extracts containing mixtures of various cyanotoxins and other cyanobacterial metabolites than of pure MC-LR and ANTX used in equivalent concentrations. The influence of cyanobacterial extracts containing natural mixtures of several cyanobacterial metabolites on zooplankton has been extensively studied (Hulot et al. 2012; Smutná et al. 2014; Barrios et al. 2015). However, only a few of these works characterised extracts for other metabolites than cyanotoxins. Our study revealed that of the two extracts tested, from *Microcystis* spp. (A) and from *P. agardhii* I (B), only the extract of *P. agardhii* was toxic to daphnids and none of them to rotifers. *M. aeruginosa*, which predominated in cyanobacterial biomass in extract A, was previously classified (Tillmanns et al. 2008 and references therein) as one of the most harmful species to zooplankton (independent of its MC production) in case of ingestion of its cells. Both extracts tested were characterised by high MC to Chl-a ratios but differed considerably in other oligopeptide’s profile. Similar high ratios were reported for some strains of *M. aeruginosa* by Lürling et al. (2017). *Microcystis* spp. in our study produced five variants of MCs, four cyanopeptolins (CPL), two anabaenopeptins (APs) and one aeruginosamide, however, other oligopeptides, reported for *Microcystis* spp. (Welker and Döhren 2006) such as microginins, microviridins and cyclamides were not detected. Czarnecki et al. (2006) reported various inhibition of trypsin-like activity in *Daphnia* caused by different strains of *Microcystis*. The effect was dependent, among others, on amino-acid composition of cyanopeptolins, but some of their variants together with microginins and microcystins had no effect on *Daphnia*.

Toxicity of cyanobacterial metabolites dependent also on the exposure route and cyanobacterial cell ingestion was reported to be more deleterious when compared to the dissolved fraction of cyanotoxins (Ferrão-Filho and Kozlowsky-Suzuki 2011). Ingestion and digestion of cyanobaterial cells is considered the primary mechanism of MC uptake by *Daphnia* (Rohrlack et al. 2005). Non-ribosomal peptide profiles in *Microcystis* spp. may essentially differ and their structure depends also on environmental factors (Tonk et al. 2009; Schwarzenberger et al. 2013b); therefore, their toxicity to hydrobionts can variate. Oligopeptide profile in the extract B of *P. agardhii* I was different than of *Microcystis* and the toxicity of the *P. agardhii* extract to *Daphnia* increased in a dose-dependent manner. *Planktothrix* spp. beside MCs, aeruginosins (AERs) and cyclic anabaenopeptins (APs) can also produce microviridins (MDN D-F, J) (Shin et al. 1996; Rohrlack et al. 2004) and cyanopeptolins (Grabowska et al. 2014) and, therefore, different sub-populations can have various oligopeptide profiles which determine their toxicity. Different APs produced by several species of cyanobacteria are potent protease inhibitors (Sivonen and Börner 2008) and in nature are as common as MCs (Rohrlack and Utkilen 2007; Kurmayer et al. 2011; Grabowska et al. 2014). Also, AERs are inhibitors of carboxypeptidase, serine proteases and (like MCs) protein phosphatases (Shin et al. 1998; Baumann et al. 2007; Kohler et al. 2014). Microcystins (inhibitors of protein phosphatases PP1 and PP2A), classified as cyanotoxins, constitute ca. 30% of oligopeptides produced by cyanobacteria (Janssen 2019 and references therein). It strongly suggests that the toxicity of cyanobacterial extracts is determined by the whole profile of the oligopeptides, not only by MCs or ANTX.

Biological role of cyanobacterial oligopeptides, which are a large group (over 700 structural variants) of mostly non-ribosomal metabolites produced by various cyanobacterial taxa (Welker and Döhren 2006; Janssen 2019 and references therein), is still unclear and under discussion. Serine proteases, such as trypsin and chymotrypsin, are the most important digestive enzymes in the gut of *Daphnia* (Von Elert et al. 2004) and occur also in some Rotifera (Hara et al. 1984); however, in rotifers, they may vary considerably between species and individuals as a result of individual differences in feeding activity, age or life stage (Štrojsová and Vrba 2005). It suggests that in natural conditions some oligopeptides may be even more harmful than MCs and ANTX to certain zooplankton species, because digestion inhibitors become active at much lower concentrations than cyanotoxins (Von Elert et al. 2004). Digestive enzyme inhibition would cause starvation and slow death, but cannot explain the acute toxicity observed within 24 h both in our experiment and previous study with a purified oscillapeptin J (Blom et al. 2006). The acute toxicity of cyanobacterial compounds have been not fully understood, yet and probably may be based on a few different modes of action. For example, MCs beside PPs inhibition caused also other biochemical alterations in zooplankton (e.g. inhibition of activity of glutathione-S-transferases and acetylocholinesterase; Ferrão-Filho and Kozlowsky-Suzuki 2011 and references therein).

The observed species-specific response in this study to the mixtures of various cyanobacterial metabolites is in agreement with some previous reports. For example, Blom et al. (2006) showed that the purified [D-Asp^3^(E)-Dhb^7^]MC-RR exerted the highest toxicity to crustaceans (*Eudiaptomus* sp. and *Daphnia* sp.) and the lowest to the rotifer *B. calyciflorus* (24 h-LC50 = 1.2, 21.9, 162.9 mg/L, respectively). The toxicity of oscillapeptin J (cyanopeptolin), an efficient inhibitor of trypsin (Baumann et al. 2007), was found only for *Eudiaptomus* sp. (24 h-LC50 = 64.7 mg/L) and *Daphnia* sp. (24 h-LC50 = 226.4 mg/L) but not for *B. calyciflorus* (Blom et al. 2006). According to Baumann and Jüttner (2008), *P. rubescens* containing mostly [Asp^3^Dhb^7^]MC-RR and oscillapeptin J caused mass mortality of *Daphnia* sp. in a Swedish lake, whereas copepods were not affected. Hulot et al. (2012) reported that the extract of the *P. agardhii* strain with unknown content of other metabolites but containing [D-Asp^3^]MC-LR, [D-Asp^3^]MC-RR and [D-Asp^3^]MC-HtyR had stronger effects on the survivorship of *Daphnia magna* Straus than the MC-free strain. However, both extracts (with MCs or MC-free) had disruptive effects on the reproduction of cladocerans. The comparison of effects of two cyanobacterial extracts with similar MC concentrations but different amounts and numbers of other oligopeptides strongly suggest that APs and AERs (produced by *P. agardhii* I) could be more toxic to *D. pulex* than MCs. Therefore, bioactive oligopeptides like anabaenopeptins and aeruginosins should be considered as the class of cyanotoxins. As it is in the case of microcystins, the production of APs and microviridins by *P. agardhii* was linearly correlated to the growth rate and biomass of the cyanobacterium (Rohrlack and Utkilen 2007), although opposite results were also reported (Kosol et al. 2009).

In one lake different strains of the same species may produce (or not) a series of different MCs and other oligopeptides (Czarnecki et al. 2006). For example, in a dam reservoir in Poland, in different years and seasons, from 7 to 22 oligopeptides other than MCs (i.e. anabaenopeptins, aeruginosins, and also single representatives of aeruginosamides, cyanopeptolins and planktocyclins) were found in *P. agardhii* (Grabowska et al. 2014). Hence, each cyanobacterial bloom can exert different toxic effects. In our study, the metabolites produced by different populations of *P. agardhii* from the same lake (but developed in different years) revealed opposite toxic effects on two zooplankton species. It is quite probable that in the most toxic extract (C) of *P. agardhii* II other oligopeptides were present than in the extract B and therefore, their toxic effects were different: extract C (more harmful to *B. calyciflorus* than to *Daphnia*) was obtained from a larger cyanobacterial biomass than extracts A and B and could contain higher number and concentrations of various oligopeptides. Interestingly, even non-MC-producing *Planktothrix* strain produced aeruginosin 828A which inhibited thrombin and trypsin in *Thamnocephalus platyurus* Packard (Kohler et al. 2014). The relative contribution and abundance of oligopeptides depend on the chemotype structure of cyanobacterial populations and may change in response to environmental factors such as water depth and light intensity (Rohrlack and Utkilen 2007; Agha et al. 2013).

Different species of *Dolichospermum* can produce a range of different toxins and non-ribosomal oligopeptides with harmful activity to aquatic organisms (Sivonen and Jones 1999; Rouhiainen et al. 2010). The extracts of *Dolichospermum* spp., containing both ANTX and MCs in different proportions (and unknown number and amount of other oligopeptides) affected rotifers and cladocerans in a dose-dependent manner. *D. lemmermannii* extract with higher total cyanotoxin concentrations was twofold more toxic to daphnids and almost fivefold more toxic to rotifers than the extract of *D. flos-aque*. On the other hand, Barrios et al. (2015) reported higher toxicity of crude extract of *Dolichospermum planctonicum* (Brunnthaler) Wacklin, L.Hoffmann and Komárek (with unknown content of toxic metabolites) to the small cladoceran *Ceriodaphnia cornuta* G.O. Sars than to the rotifer *Plationus patulus* (Müller). It suggests that successive mass development of different species/strains of cyanobacteria with complex composition of metabolites causes a real but various threat for different taxonomic groups of zooplankton.

Generally, our results suggest that secondary cyanobacterial metabolites other than cyanotoxins such as MCs, ANTX or their synergistic interactions contribute to the toxicity observed in daphnids and rotifers. As was reported by Freitas et al. (2014), a mixture of two cyanobacterial extracts, one containing MC-LR, the second one anatoxin-a(S), revealed both additive and synergistic toxicity to feeding rate of *D. magna*. However, profiles of other metabolites in the extracts were unknown and their influence can not be excluded. It seems that interactions between cyanobacterial metabolites (present in water bodies both intra- and extracellular) and zooplankton may vary between species or genera of the planktonic invertebrates, and in nature they may also depend on environmental factors or the efficiency of detoxification processes, which in the case of MCs rely on the production of MC-GSH conjugates (Wojtal-Frankiewicz et al. 2013) or involvement of cytochrome P450 in the case of ANTX (Wiegand and Pflugmacher 2005). Blom et al. (2006) reported adaptation of cladoceran *Daphnia* and copepod *Eudiaptomus* to two intracellular metabolites (oscillapeptin J and a derivative of MC-RR) present in cyanobacterial diet as a consequence of prolonged exposure to cyanobacterial blooms; however, there is no information concerning rotifers and detoxification processes related to other cyanobacterial metabolites.

## Conclusion

Summing up, the results presented here suggest that natural mixtures of cyanobacterial metabolites (including oligopeptides other than MCs) can be an important selective factor influencing zooplankton communities. Not only concentrations but also a profile of oligopeptides may determine toxicity of cyanobacteria. The resistance of *B. calyciflorus* to pure cyanotoxins (MC-LR and ANTX) was higher than that of *D. pulex*; however, no evidence for higher resistance of rotifers than daphnids to different mixtures of cyanobacterial metabolites was found. Therefore, different cyanotoxins and other bioactive metabolites, such as oligopeptides, should be accessed as they may pose a risk to aquatic organisms, not only acting in a separated but in an additive or synergistic manner. In addition, each mass development of cyanobacteria, even the ones not producing MCs or ANTX, should be considered as potential threat to zooplankton communities.
